# A Survey of Zone II Flexor Tendon Repair Techniques and Rehabilitation Protocols Preferred by Malaysian Orthopaedic Practitioners

**DOI:** 10.5704/MOJ.2207.011

**Published:** 2022-07

**Authors:** A Shalimar, CH Lim, SK Wong, SY Lau, FA Anizar, S Shukri

**Affiliations:** 1 Department of Orthopaedics and Traumatology, Universiti Kebangsaan Malaysia, Kuala Lumpur, Malaysia; 2 Department of Orthopaedic, Hospital Alor Setar, Alor Setar, Malaysia; 3 Department of Orthopaedic, Hospital Sarikei, Sarikei, Malaysia

**Keywords:** flexor tendon, core suture, modified Kessler, Adelaide, epitendinous

## Abstract

**Introduction:**

Flexor tendon repair is challenging mainly due to the need to balance between a strong repair technique, ease of tendon gliding and early mobilisation to prevent adhesions while preventing tendon rupture. While different countries have different preferences in repair techniques, core sutures and suture types, there is still no study in Malaysia regarding our preference and whether we are following the current evidence.

**Materials and methods:**

We performed a survey with a standard questionnaire distributed during our annual national orthopaedic meeting in 2019. The standard questionnaire consisted of 24-objective multiple-choice questions concerning the treatment of flexor tendon injury were distributed with consent. A total of 290 questionnaires that were filled out correctly were included in this study.

**Results:**

The majority of respondents preferred the Modified Kessler technique (n=96, 33.1%) followed by the Adelaide technique (n=81, 27.9%) and Double Modified Kessler (n=45, 15.5%). However, for the number of core strands in the repair, the majority utilised the 4-strand (n=203, 70%), followed by 2-strand (n=34, 11.7%) and 6-strand (n=21, 7.2%). The majority utilised Prolene sutures (n=259, 89.3%) with a suture size of 4/0 (n=157, 54.1%). For rehabilitation, 56.9% (n=165) preferred early passive motion, 27.6% (n=80) early active motion and 14.8% (n=43) would strictly immobilise.

**Conclusion:**

There is still no consensus as to the best technique; however, the aim of tendon repairs is still the same around the world. It would be helpful to know our preferences to improve our current practice and outcomes following these common flexor tendon injuries in hand.

## Introduction

Flexor tendon repair is challenging mainly for the postoperative management has to be balanced as mobilisation prevent adhesions and improve gliding, yet, risks tendon rupture^1^. The flexor tendon also needs to glide through a narrow, constrictive tendon sheath and any repair which is bulky may result in limited motion^1^.

Strickland^2^ described an ideal primary flexor tendon repair should comprise easily placed in tendon, secure knots, smooth junctions, minimal gapping, minimal interference with tendon vascularity and sufficient strength throughout healing to permit early range of motion. Early active mobilisation is important in preventing formation of adhesions while stimulating tendon healing^3^. However, immediately after tendon repair, the strength of the repair itself is dependent on the suture material and the technique used.

Current evidence shows the ideal core suture material should have a high tensile strength, inextensible, cause no tissue reaction and easy to handle and knot^4^. Increasing the size of the core suture increases the strength of the repair^4^.

Different countries have different surgeon preferences. In UK, the preferred technique is two strand Kessler repairs5, with prevalence of 36%, despite it being biomechanically inferior to a four or more-strand repair. The most commonly used sutures are Prolene (Ethicon, Edinburgh), Ethibond (Ethicon) and Ticron [Tyco Healthcare, Gosport, UK]^5^.

Currently in Malaysia, we do not have data on the current practice preferences by orthopaedic surgeons and young medical officers in orthopaedics. This study aims to identify their surgical preferences in ensuring the quality of flexor tendon repair and its outcome.

## Materials and Methods

Ethical approval was obtained from the Ministry of Health with an approval code of NMRR-17-3424-39243 (IIR). A standard questionnaire was distributed during the 49th Malaysian Orthopaedic Association Annual Scientific Meeting 2019 held in Hilton Hotel, Kuala Lumpur, Malaysia, in 2019. Any participant of the conference was invited to participate in the study. The standard questionnaire consisted of 24-objective multiple-choice questions concerning the treatment of flexor tendon injury were distributed with consent. A total of 290 questionnaires that were filled out correctly were included in this study. A total of 60 questionnaires were excluded from the study.

## Results

Demographically, majority of the respondents were aged 3040 years (160, 55.2%), followed by <30 (101, 34.8%), 41-50 (18, 6.2%), and >50 (11, 3.8%). Out of 290 respondents, 222 (76.6%) practised as orthopaedic medical officers, 57 (19.7%) orthopaedic surgeons, 9 (3.1%) hand surgeons and 2 (0.7%) hand fellows. In terms of place of practice, 221 (76.2%) work in governmental hospital, 43 (14.8%) in university hospital and 26 (9.0%) in private hospital.

Approximately a third (110, 37.9%) did 6-10 flexor tendon repair cases the previous year, followed by (76, 26.2%) 0-5 cases, (54, 18.6%) 11-15 cases, and (50, 17.2%) more than 15 cases. Almost half of the respondents (141, 48.6%) preferred general anaesthesia, followed by regional anaesthesia (48, 16.6%), local anaesthesia (35, 12.1%), Wide Awake Local Anaesthesia No Tourniquet (WALANT) (29, 10.0%) and the remaining (37, 12.8%) had more than one preferred anaesthesia technique. 80% of them utilised tourniquets in flexor tendon repair, whilst 16.2% did not use a tourniquet in surgery.

**Fig 1:**
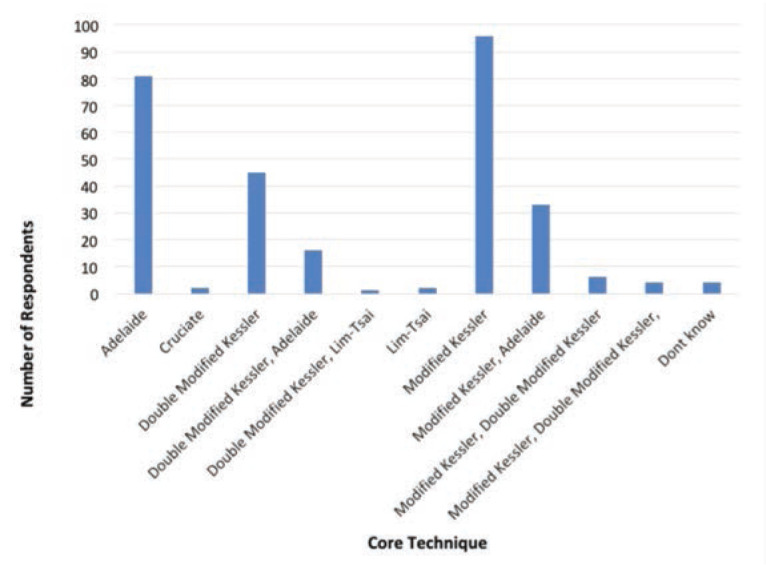
Preferred core technique of respondents in flexor tendon repair.

**Fig 2:**
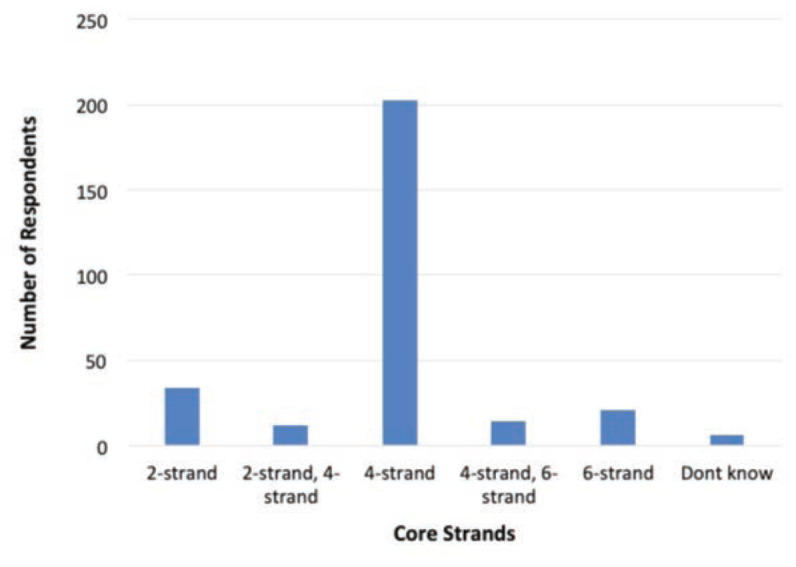
Preferred core strand of respondents in flexor tendon repair.

**Fig 3:**
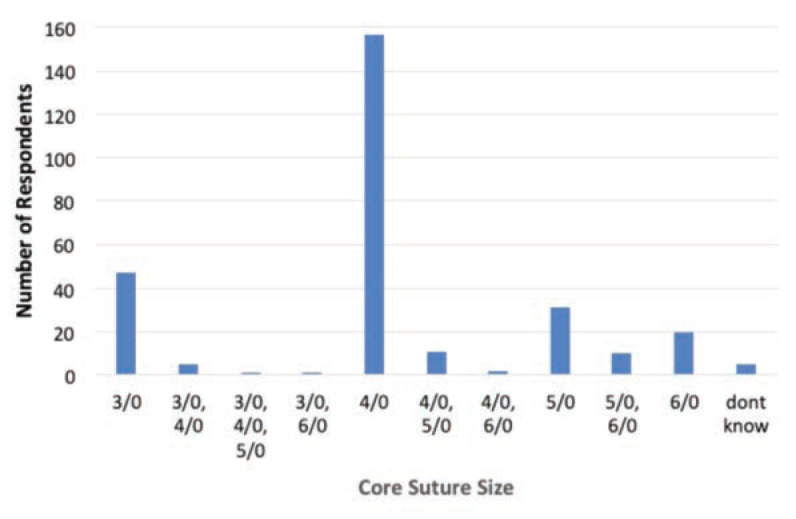
Preferred core suture size of respondents in flexor tendon repair.

As for the repair technique, the top three core techniques of flexor tendon repair were Modified Kessler (n=96, 33.1%), Adelaide (n=81, 27.9%) and Double Modified Kessler (n=45, 15.5%) as shown in Fig. 1. For the number of core strands in the repair, the majority utilised the 4-strand (n=203, 70%), followed by 2-strand (n=34, 11.7%), 6-strand (n=21, 7.2%), and other preferences are as shown in Fig. 2. The top three suture size used were size 4/0 (n=157, 54.1%), 3/0 (n=47, 16.2%) and 5/0 (n=31, 10.7%). For suture types, majority of the respondents preferred Prolene (n=259, 89.3%), followed by Supramid (n=9, 3.1%) and Ethibond (n=8, 2.8%) as shown in Fig. 3.

As for epitendinous repair, 83.8% (n=243) of respondents chose to do an epitendinous repair, 13.1% (n=38) do not perform an epitendinous repair, and 3.1% did not know-how. The top suture size for the epitendinous repair was 6/0 (40.3%), followed by 5/0 (27.2%), 4/0 (23.9%), and 3/0 (5.3%). Among those who did epitendinous repair, 89.7% (n=218) preferred Prolene as the suture type of epitendinous repair, followed by Ethibond (7, 2.9%), Ticron (5, 1.6%), and others.

For Zone II flexor tendon injury, 36.2% (n=105) would repair flexor digitorum profundus (FDP) tendon only, 32.4% (n=94) would repair both FDP and flexor digitorum superficialis (FDS), 24.1% (n=70) would repair FDP with a slip of FDS, and 7.2% (n=21) did not know. These are followed by an open-ended question that requires the participant to answer the rationale behind their repair techniques.

The justification for repairing FDP only includes: because Zone II is “no man’s island”, that area is narrow, it would be a bulky repair if both FDP and FDS were repaired, to have better tendon gliding, allow smooth tendon excursion, better strength, sufficient for function, to reduce adhesion and to prevent stiffness, for early range of motion, and to ease rehabilitation. Some mentioned it is due to their own preference and familiarity or that they were trained as such. A few others said repairing FDP alone is technically less demanding; FDS repair requires microscopic anatomy restoration and require more skills.

For those who choose to repair both FDP and FDS, their reasons were both FDP and FDS are essential. By repairing both tendons, it provides more strength and better function, early mobilisation and rehabilitation. Some believe stiffness can be avoided if the repair were done correctly with good post-op rehabilitation. For those repairing FDP with a slip of FDS, their reason is mainly to prevent adhesions. A few respondents felt it gives the best functional outcome as it is a well-studied repair, but others did so because of training and familiarity.

For rehabilitation, 56.9% (n=165) preferred early passive motion, 27.6% (n=80) early active motion, 14.8% (n=43) strictly immobilise and 0.7% (n=2) did not know. As for duration of strict immobilisation, up to two weeks was the top preference (n=199, 68.6%), followed by up to six weeks (n=85, 29.3%), more than six weeks (n=2, 0.7%) and four weeks (n=1, 0.3%). 1% (n=3) did not know the duration of strict immobilisation. While for type of strict immobilisation, the majority (n=138, 47.6%) selected a dorsal slab as their preference, followed by thermoplastic splint (n=103, 35.5%), fiberglass slab (n=13, 4.5%), POP dorsal slab and thermoplastic splint (n=13, 4.5%).

As for protective immobilisation, up to six weeks was the top preference (n=177, 61.0%), followed by up to two weeks (n=99, 34.1%), more than six weeks (n=8, 2.8%) and four weeks (n=1, 0.3%). 1.7% (n=5) did not know the duration of strict immobilisation. For type of protective immobilisation, the majority (n=150, 51.7%) selected thermoplastic splint as their preference, followed by POP dorsal slab (n=80, 27.6%), fiberglass slab (n=18, 6.2%), commercial splint (n=11, 3.8%).

For commencement of active motion, mostly preferred at six weeks (n=110, 37.9%), followed by at four weeks (n=80, 27.6%), at two weeks (n=72, 24.8%) and immediate (n=26, 9.0%). 0.7% (n=2) did not know. For commencement of passive motion, mostly preferred at two weeks (n=122, 42.1%), followed by immediate (n=90, 31.0%), at four weeks (n=41, 14.1%) and at six weeks (n=32, 11.0%). A total of 1.7% (n=5) did not know. Almost half (n=138, 47.6%) would refer the patients to both physiotherapist and occupational therapist, followed by occupational therapist only (n=106, 36.6%), physiotherapist only (n=44, 15.2%), and 0.7% (n=2) did not know which is the suitable reference.

## Discussion

In this study, the flexor tendon injury repairs were mainly done by orthopaedic medical officers aged 30-40 years and practising in government hospitals who have varying rotations in orthopaedic hand unit at different frequency and intervals and different hospitals with different standard of care. Most of them performed 6-10 flexor tendon repairs in the previous year.

Interestingly, those who refused to participate in this study were mainly above 40 years old, consultant level practising in other subspecialities apart from hand surgery or worked in private hospitals where they rarely or never perform flexor tendon repairs. Due to unfamiliarity with the latest repair technique and rehabilitation of flexor tendon injuries, they usually refer the cases to hand surgeons or government hospitals for better management.

For the anaesthesia technique for flexor tendon repair, we observed that almost half of the respondents preferred general anaesthesia. GA is the most widely used anaesthetic technique for ambulatory surgery^6^. It is probably because the anaesthesiologists are more familiar in giving GA^7^. Compared with the older GA agents, short-acting GA agents produce significantly fewer adverse effects, a better recovery profile, higher patient satisfaction, and more cost-effective^8^. A prospective randomised study showed that RA did not improve pain control at home up to 14 days after ambulatory hand surgery. Still, RA improved early pain control with fewer adverse effects and earlier hospital discharge^9^.

A meta-analysis had demonstrated it is probably impossible to differentiate the mortality between GA and RA. Still, RA does offer better analgesia and a significant reduction in post-operative pain^10^. Another meta-analysis had shown that although RA increases the induction time, RA reduces the post-operative pain and the need for postanaesthetic care unit analgesics. Still, it does not lessen the ambulatory surgery unit time^11^. Patients undergoing RA for hand surgery are less likely to need analgesic and antiemetic medication during the recovery period than GA^12^.

Applying a forearm or arm tourniquet to provide better visibility of the surgical field is a common and universally accepted practice of hand surgery. LA is often injected into the operative site to compensate for the pain generated by the applied tourniquet. Thus, there is a significant increase in the use of pure local anaesthesia for hand surgery for the past ten years. Although it is not that popular among the respondents in this study, this trend is likely to continue to increase given the new techniques to decrease the pain of local anaesthesia injection and excellent cost saving of tourniquet-free pure local anaesthesia^13^.

Most hand surgeries were performed with a tourniquet to provide a bloodless surgical field. In recent years, many hand surgeons are moving away from the traditional surgery using a tourniquet and sedation to WALANT as the long-held belief of epinephrine causing finger necrosis has been disproved^14-16^. The strategic use of LA with epinephrine is safe in flexor tendon repair, and it allows intra-operative control of overall motion and function^17^. Although only 10% of respondents preferred WALANT in our study, we expect there will be a significant increase in the application of WALANT among the surgeons in the future with more and more scientific evidence regarding WALANT benefits, and training conducted.

In the literature, there is no consensus on the ideal flexor tendon repair technique. The principal aim of the flexor tendon repair is to provide a strong healing tendon that can withstand the early active rehabilitation programmes. Many studies have proven that it is essential to use a reliable and robust suturing technique to do the primary repair of a divided tendon, to minimise the failure rates regarding the complications like rupture or gap formation^18-20^.

Modified Kessler was the most popular core technique of flexor tendon repair in our study, followed by Adelaide and Double Modified Kessler. The popularity of modified Kessler is aligned with another study done in the United States^21^. It is probably because modified Kessler is relatively simple compared with other techniques. A meta-analysis demonstrated adhesion development is 57% lower when the modified Kessler technique is used^22^.

A biomechanical analysis demonstrated Adelaide repair is a reliable suture technique and better than other techniques, including Modified Kessler, Lahey, and Becker due to its only small displacement, high stiffness and almost no suture pull-out^23^. Despite of its biomechanical strength, Adelaide repair has its disadvantages, too, include exposed suture on the surface of the tendon, increased tissue handling from placing the cross-locks, and the need to ensure tendon ends are well approximated and the additional tensioning of the repair cannot be easily achieved at the time of final knot tying^24^.

A study has demonstrated 2-strand repair had significantly greater gap formation compared with 4- and 6-strand repairs, while 6-strand had substantially higher tensile strength than 2- and 4-strand methods. However, the 6-strand repair is potentially associated with more adhesion formation than 2-and 4-stand methods due to extensive tendon manipulation resulting in more exposed sutures on the tendon surface^25^. A rabbit model demonstrated 4-strand core suture improved flexor tendon repair compared with the 2-strand repair^26^.

In fact, the size of the core suture has an impact on tensile strength and work of flexion of the repair, from 4/0 to 2/0 increased maximum tensile strength but also resulted in increased work of flexion^27^. Thus, the surgeons need to balance the tensile strength and work of flexion.

Most of the respondents (89.3%) used Prolene. A study has shown all the polyester fibre-based sutures performed exceptionally well, with Mersilene proving to be the best overall, but no statistically different compared with Ticron and Ethibond.

Prolene performed better than Supramid but still developed a significant amount of creep with a high failure rate. Supramid performed extremely poor during both static and cyclical testing in the study. The study suggested that suture material itself played a vital role in the eventual outcome of flexor tendon repair^28^.

A meta-analysis of 29 studies showed that core suture technique or the use of an epitendinous suture does not influence the rupture rate. However, the presence of an epitendinous suture does reduce the rate of re-operation by 84%^22^.

Epitendinous-first flexor tendon repair significantly reduced the mean gliding resistance, eased the placement of core sutures and caused decreased bunching^29^. Sue *et al* recommended a 6/0 polypropylene suture to be used for the epitendinous suture^30^. It is aligned with our study in which the preferred suture size for epitendinous repair was 6/0 and the suture material was Prolene.

Until today, the method of Zone II flexor tendon repair is still controversial. Repair of both tendons in Zone II is ideal, but it is technically demanding^1^. Repair of the FDP tendon alone is good and less technically demanding. Still, it carries a risk of failure if the repaired tendon breaks down during physiotherapy or if there is a breakage of the suture line^1^. Most hand surgeons preferred to repair the FDP and a slip of FDS as repairing both slips of FDS would result in overcrowding within the sheath and pulleys, which compromised the outcome^31^.

There is a debate for more than 75 years regarding postoperative management of flexor tendon injuries such as type of rehabilitation, type of splint and its duration, and commencement of motion. Two recent meta-analyses have shown that early active mobilisation regimes give better functional outcomes but with a higher risk of rupture^32,33^. Conversely, a systemic review provides moderate to strong evidence that place and hold exercises offer better outcomes than passive flexion protocols for patients with 2- to 6-strand repairs. However, at this moment, there is no sufficient evidence to support the proper active motion for flexor tendon rehabilitation^34^. Although the rehabilitation has evolved from passive motion protocols to early active motion, most of our respondents still preferred early passive motion.

Still, there is no clear consensus in the literature on whether we should refer the patients to an occupational therapist or physiotherapist for rehabilitation. In general, the surgeons would refer the patients to a hand therapist, but the hand therapist can be either from an occupational therapist (OT) or physiotherapist (PT) background. It depends on the hand therapy practice profile in that country. In the UK and US, hand therapists predominantly are from an OT background. Whereas in South Africa, hand therapists are predominantly from a PT background. In Canada, the ratio of OT to PT to become hand therapists is more diminutive than 1:1^35-37^. Our study indicates that almost half of respondents preferred to refer the patients to both occupational therapist and physiotherapist, followed by an occupational therapist only and lastly, physiotherapist only.

In short, successful rehabilitation of flexor tendon injuries is a complex process that requires numerous clinical decisions by the occupational or physiotherapist over a 12- to 16-week period. However, a study demonstrated that most therapist autonomy was perceived to be low in the clinical decision. Most therapists can freely set the frequency of the rehabilitation sessions but not choose the type of protocol to be used and the initiation timing of rehabilitation. The shared decision between therapist and surgeon frequently occurred; however, surgeons generally have more autonomy regarding the key elements of rehabilitation than the therapists^38^. It is something that we can investigate in the future.

Limitations of this study are that the questions were multiple-choice, and those who selected the answers may not necessarily be practising the selection. They may just have heard of the technique or method, and some may choose blindly. A pilot study was done during a local tendon workshop on 15 participants however the results were not available, and the questionnaire was not pre-validated. Hence, the strength of the study is low.

We recommend that the Malaysian Society for Surgery of the Hand (MSSH) produce a consensus paper on the recommended flexor tendon repair techniques and a rehabilitation protocol. This should be made available on the MSSH website together with educational resources. Currently, there are approximately 3-4 tendon workshops held yearly, and this should be continued or perhaps increased in frequency.

## Conclusion

Our study showed various preferences of flexor tendon repair techniques and rehabilitation protocol mostly as knowledge passed down from seniors rather than evidence based. We recommend that a national guideline is developed as a standard protocol for all practising orthopaedic surgeons or medical officers.
